# The Role of GSK3β in T Lymphocytes in the Tumor Microenvironment

**DOI:** 10.3389/fonc.2020.01221

**Published:** 2020-07-24

**Authors:** Anastasios Dimou, Konstantinos N. Syrigos

**Affiliations:** ^1^Division of Medical Oncology, Mayo Clinic, Rochester, MN, United States; ^2^Division of Medical Oncology, Third Department of Medicine, University of Athens, Athens, Greece

**Keywords:** GSK3β, Tregs, IL-10, NFAT2, PD-L1

## Abstract

Immunotherapy options for patients with cancer have emerged following decades of research on immune responses against tumors. Most treatments in this category harness T cells with specificity for tumor associated antigens, neoantigens, and cancer-testis antigens. GSK3β is a serine-threonine kinase with the highest number of substrates and multifaceted roles in cell function including immune cells. Importantly, inhibitors of GSK3β are available for clinical and research use. Here, we review the possible role of GSK3β in the immune tumor microenvironment, with goal to guide future research that tests GSK3β inhibition as an immunotherapy adjunct.

## Introduction

It is a truism that non-synonymous mutations bear the potential to generate neoantigens and elicit immune reactions against tumors (1). Therefore, development of established tumors requires evasion of host immune defenses (2). Further, the complex interaction between the immune system and the tumor takes the steps of elimination, equilibrium, and escape successively and largely reflects tumor evolution under the selective pressure of immunosurveillance (3, 4). Immune evasion is multifaceted and currently considered one of the hallmarks of cancer. The presence of immune infiltrates in the microenvironment of tumors is highly relevant from a therapeutic perspective, illustrated by multiple approvals of drugs which exert anti-cancer activity by harnessing the immune system across tumor types.

The main arms of immunity are the innate and adaptive responses against pathogens. The former is a faster type of response, does not require priming and does not exert target specificity which are all features of adaptive immunity (5). Innate immunity depends mostly on natural killer cells (NK cells), dendritic cells, macrophages and polymorphonuclear (PMN) cells. These, recognize pathogen-associated molecular patterns (PAMPs) and damage-associated molecular patterns (DAMPs) (6). PAMPs are pathogen derived and DAMPs are associated with injury and inflammation. Conversely, the cell types that orchestrate adaptive immunity are mainly the B and T lymphocytes. It is noteworthy that for both innate and adaptive responses, several cell types might adopt an immunosuppressive role in a context dependent manner, as is often the case with neutrophils (7), macrophages (8), and T regulatory cells (Tregs) in cancer (9).

In the tumor microenvironment, the main lymphocyte populations include either CD4 or CD8 positive cells. The former, use their T cell receptor (TCR) to recognize peptides bound to human leucocyte antigen (HLA) class II in the cell surface of antigen presenting and tumor cells, whereas the latter recognize peptides bound to HLA class I molecules in the plasma membrane of tumor cells. CD4 positive, or “helper” T lymphocytes (Th) further divide into Th1, Th2, and Th17 subgroups under the influence of cytokine and chemokine cues (10). Their main role is to prime B lymphocyte activation (11, 12) and antigen presentation to CD8+ cells (13) whereas they can also function as cytotoxic T lymphocytes (CTLs) (14). Conversely, CD8 positive T lymphocytes mainly function as CTLs. Finally, Tregs can either develop independently as natural Tregs (nTregs) or emerge from CD4 positive T cells, as inducible Tregs (iTregs).

In a broad schema, the cytotoxic T lymphocytes (CTLs) can either be excluded from the tumor core (“cold” tumors), infiltrate the tumor core (“hot” tumors), or finally form immune aggregates that resemble secondary lymphoid organs (15). In the first scenario, an immunosuppressive microenvironment prevents the CTLs entry in the tumor core. In “hot” tumors, immune check points like the PD1-PDL1 axis play a central role in immune evasion. Finally, this system is highly regulated by interplay between cancer related mutations, the cytokine and chemokine milieu and importantly activation of molecular pathways in the adaptive immune system cells.

Glycogen Synthase Kinase 3β (GSK3β) is a serine threonine kinase with multiple substrates that was originally described for its role in the synthesis and storage of glycogen in skeletal muscle (16). Lithium, a GSK3β inhibitor, has been used for decades for the treatment of bipolar disorder (17). More recently, GSK3β is studied as a promising target for anti-cancer treatment with supporting preclinical data in a number of malignancies, including pancreatic cancer, colon cancer, bladder cancer, kidney cancer, and melanoma (18). GSK3β is very rarely altered at the genomic level in cancers, therefore pharmaceutical inhibition of GSK3β is not expected to be specific for cancer cells. With clinical trials of GSK3β inhibitors under way in the phase I space, an important question moving forward is the clinical effect of these drugs on cell types other than the cancer cells. Importantly, a growing body of literature shows that GSK3β inhibitors modulate the immune system in autoimmune and neoplastic disease contexts.

The seminal finding that GSK3 beta controls PDL1 levels indicates a possible effect of GSK3 beta inhibition on the immune reaction against tumors (19). Herein, we shall review the preclinical data on the effects of GSK3 beta inhibition in the tumor immune microenvironment with focus on T lymphocytes related adaptive immunity.

## Description of the GSK3β Function

**Figure 1:**
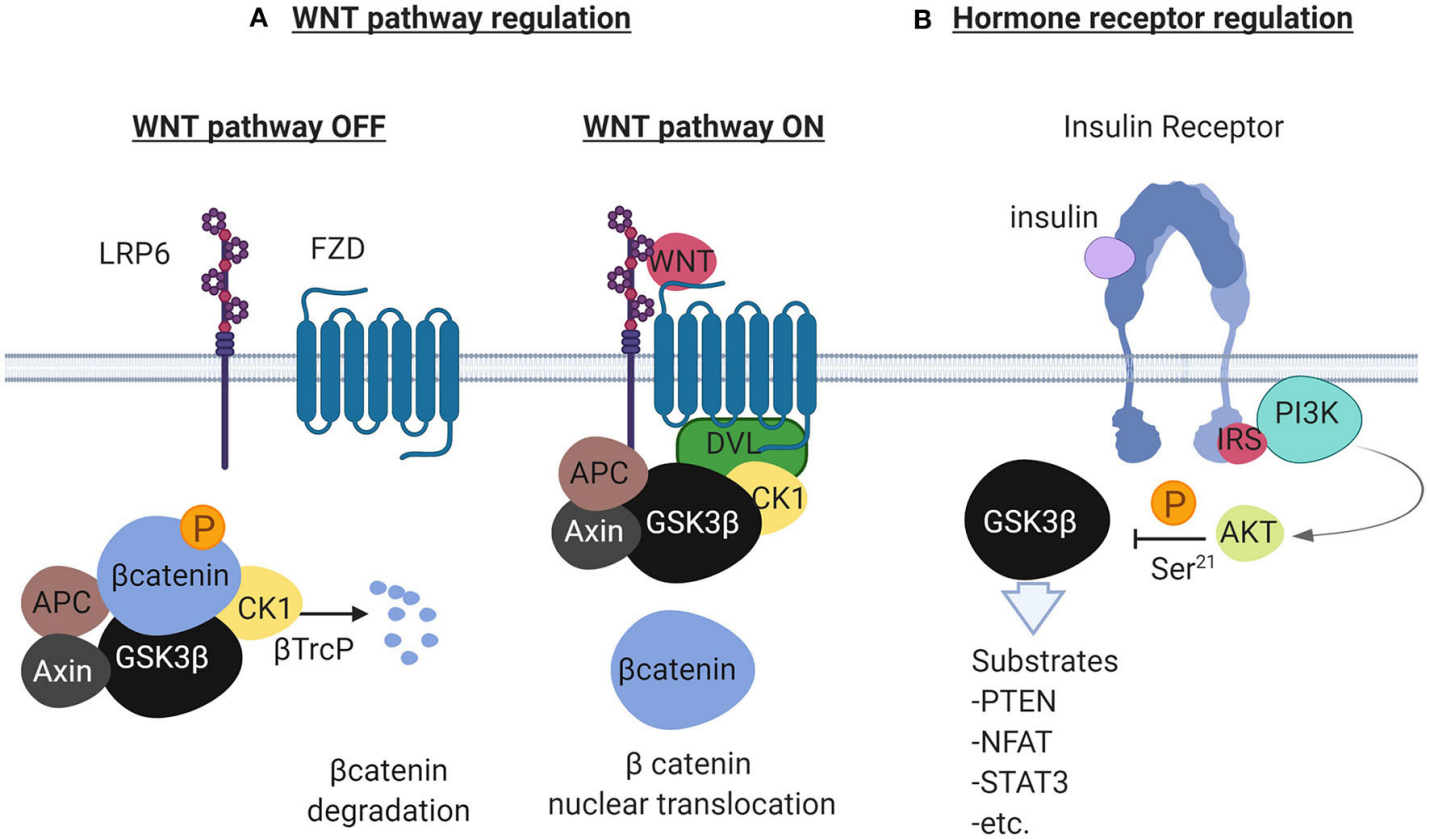
Description of the GSK3β to regulate the WNT pathway and the Receptor Tyrosine Kinase pathway illustrated here by the insulin receptor. In **(A)**, in the absence of WNT ligands (left), the scaffolding protein Axin forms a complex with beta catenin, Adenomatous Polyposis Coli (APC), GSK3β and Casein Kinase 1 (CK1). GSK3β phosphorylates beta catenin in the complex and ubiquitination by the ubiquitin ligase βTrcP follows. Ubiquitinated beta catenin is then degraded by the proteasome. In the presence of WNT ligands (right), the WNT receptors Low Density Lipoprotein Receptor related protein 6 (LRP6) and Frizzled (FZD) recruit Disheveled (DVL) that binds to the complex and separates beta catenin. Beta catenin in this condition translocates to the nucleus to activate WNT responsive genes. In **(B)**, the insulin receptor (other RTKs also), activates the PI3K/AKT pathway in the presence of insulin. AKT negatively regulates GSK3β by phosphorylating GSK3β at Ser-21. GSK3β in this cellular pool is constitutively active and is inactivated in the presence of growth factors. Created with BioRender.

GSK3 beta is a master serine/threonine kinase in the cell with more substrates than any other kinase (20). It was originally described to phosphorylate and inactivate glycogen synthase in skeletal muscle, an effect reversed by insulin (21, 22). Insulin and other growth factors, bind to cognate receptors and activate AKT which then phosphorylates GSK3β at Ser9 (23). Phosphorylation at this site inhibits GSK3β which adopts a “closed” inactive structure (24). Alternatively, G-protein coupled receptors raise the levels of cyclic AMP and activate protein kinase A which interacts with GSK3β and induces the inhibitory phosphorylation at Ser9 (25). Additionally, GSK3β has been studied as a negative regulator of the WNT pathway as it phosphorylates and induces degradation of beta catenin (23, 26). GSK3α is a significant isoform with overlapping but also some distinct functions (27). The GSK3β mode of function is unique among other kinases because it requires phosphorylation priming of the substrate at a serine or threonine position 4 amino-acids away from the GSK3β serine/threonine phosphorylation site, toward the carboxylic terminus of the substrate (28). Among the most studied substrates of GSK3β are the phosphatase and tensin homolog (PTEN) (29), cAMP responsive element binding protein (CREB) (20), beta catenin (26), and the nuclear factor of activated T cells (NFAT) (20). Figure 1 describes the main functions of GSK3β in the context of the WNT and the insulin receptor pathways.

## Effects of Lithium on Lymphocytes

Lithium is a GSK3β inhibitor that has been used for a long time in the treatment of patients with bipolar disorder. It is known from early studies that lithium decreases peripheral lymphocyte count and in reverse, increases the peripheral neutrophil count in patients (30). The thymus of mice treated with lithium chloride has smaller size and decreased cellularity related to loss of T lymphocytes (31). *In vitro*, lithium reduces lymphocyte proliferation but also inhibits their apoptosis (32). Additionally, lymphocyte function in response to various stimuli is enhanced following exposure to lithium. Little is known about the mechanistic basis of these effects. It is currently controversial whether primarily GSK3β, or other lithium targets account for the effects of lithium on lymphocytes.

## Inhibition of GSK3 Enhances Co-Stimulation During Antigen Presentation

Naïve helper (CD4 positive) T lymphocytes and to a lesser extent, naïve CTLs (CD8 positive) require three signals for their activation by APCs. TCR binding to peptide presented by the MHC II complex on an APC provides signal 1, whereas signal 2 requires binding of CD28 expressed in the T lymphocyte to B7-1 and B7-2 expressed in the APC (33, 34). Cytokines provide signal 3. Although CD28 was originally described as the main co-stimulation molecule in the immunological synapse, other molecules exist and are grouped in two main families, the immunoglobulin like (Ig-like) and tumor necrosis factor receptor like (TNFR-like) (35). Some members of the families like CD28 deliver stimulatory while others, like CTLA-4 deliver inhibitory signals (35).

**Figure 2:**
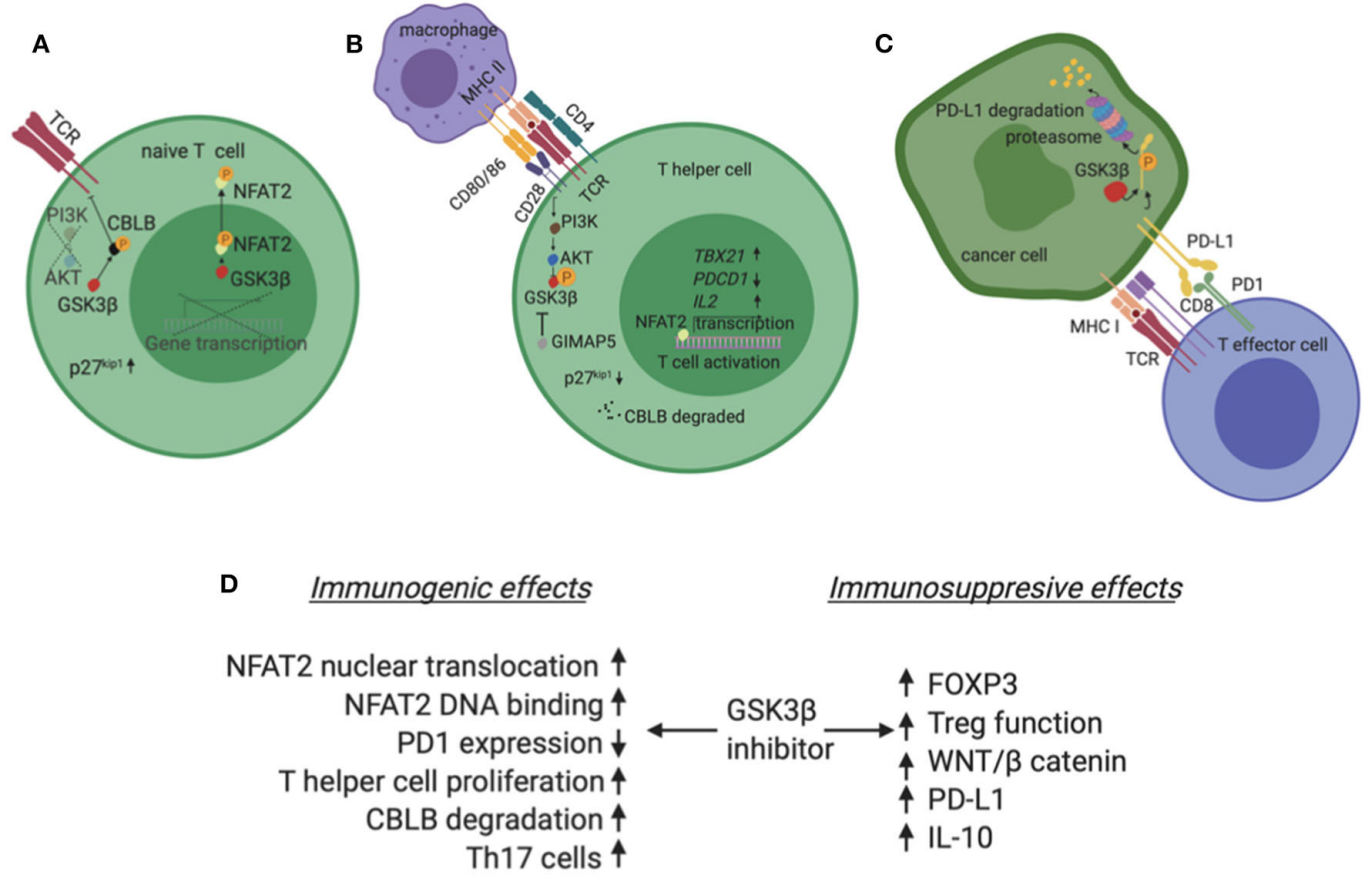
**(A)** In the absence of co-stimulation, typically in a T lymphocyte that has not been exposed to antigen yet (naive T cell), GSK3β is constitutively active and phosphorylates NFAT2. Phosphorylated NFAT2 exits the nucleus and does not induce expression of target genes. Additionally, GSK3β phosphorylates CBLB, an inhibitor of TCR. Phosphorylated CBLB is protected from degradation. **(B)** In the presence of co-stimulation, typically when an APC presents a peptide to a CD4+ T cell, the second signal initiated by CD28 activates the PI3K/AKT pathway which phosphorylates and inactivates GSK3β. In these conditions, NFAT2 is not phosphorylated, enters the nucleus and induces transcription of genes like *TBX21* and *IL2* which promote T cell activation and proliferation. Also, CBLB is degraded in a de-phosphorylated state and no longer inhibits TCR. Finally, the GTPase GIMAP5 can independently inhibit GSK3β. **(C)** PD-L1 is phosphorylated by GSK3β and marked for degradation in the proteasome of cancer cells. **(D)** Summary of GSK3β inhibitor effects in the tumor immune microenvironment highlighting both immunogenic and immunosuppressive possibilities. Created with BioRender.

CD28 signals primarily through the PI3K/AKT pathway and when activated, this axis suppresses GSK3 (Figure 2B) (36, 37). Interestingly, inhibition of GSK3 substitutes for CD28 activation in enhancing T cell proliferation (38). Mechanistically, GSK3 is constitutively active, phosphorylates NFAT transcription factors and enhances NFAT nuclear export (Figure 2A) (39). CD28 activation phosphorylates and inactivates GSK3, hence NFAT enters the nucleus in a de-phosphorylated state and induces T cell proliferation. In addition to enhancing NFAT nuclear translocation, GSK3 inhibition augments binding of NFAT2 to DNA (40). Alternatively, PI3K/AKT activation by CD28 activation/GSK3β inhibition and concurrent MAPK activation by TCR, are required to reduce the levels of p27^kip1^, a negative cell cycle modulator. The net effect is hyperphosphorylation of retinoblastoma protein, progression of cell cycle and proliferation of primary T lymphocytes (36). Beyond NFAT and p27^kip1^, CBLB, an E3 ubiquitin ligase with known inhibitory role in T cell activation, is a GSK3 substrate (41). Inactivation of GSK3 leads to reduced levels of Cbl-b and enhancement of T cell activation and autoimmunity in murine models (Figure 2B). The central role of GSK3 downstream of CD28 is also impactful in the immune tumor microenvironment. In a lymphoma model, Taylor et al. showed that GSK3 inhibition substitutes for CD28 activation and suppresses the PD1 axis (38).

GSK3β inhibition causes NFAT2 to accumulate in the nucleus of T cells and increase proliferation of T cells as well as to support maximal interleukin 2 production (42). Particularly, inactivation of GSK3β by the GTPase of immunity-associated protein 5 (GIMAP5) increases proliferation of CD4+T cells in autoimmune models (Figure 2B) (43). Conversely, Gattinoni et al. showed that GSK3β inhibition promotes the formation of a special population of T memory cells with stem cell properties (44). These cells have reduced proliferation rates and exert multipotency. Importantly, they expand fast and are more effective compared to other memory cell subsets when challenged by antigen and function against tumors.

## GSK3β Regulates the PD1/PDL1 Axis in Cancer Cells Interacting With T Effector Lymphocytes

Tumor cells express PDL1 in their cell surface which interacts with PD1 in CD8 positive T cells (45, 46). The PD1/PDL1 axis causes T cell exhaustion and is a mechanism of immune escape for tumors. Interestingly, PD1 expression defines the population of tumor reactive CD8 positive T lymphocytes with neoantigen specificity (45). Exhausted T cells retain some anti-tumor activity but do not control tumors efficiently. The benefit of drugs with anti-PD1 and anti-PDL1 activity in a subgroup of patients with a range of malignancies highlights the importance of this molecular checkpoint (47).

PDL1 is a substrate for GSK3β. This interaction leads to a number of phosphorylation steps and finally ubiquitination and proteasome degradation of PD-L1 (Figure 2C) (48). In the presence of growth factors, like Epidermal Growth Factor, AKT phosphorylates and inactivates GSK3β. In these conditions, GSK3β no longer phosphorylates PDL1 and the levels of the latter molecule rise in the cancer cell. Additionally, N-glycosylation of PDL1 further protects PDL1 from GSK3β dependent phosphorylation in breast cancer, melanoma and colon cancer models (19). Conversely, on the CD8 positive T cell, GSK3α/β induces the transcription of *pdcd1* that codes for murine PD1 in murine melanoma and lymphoma models (49). Interestingly, *ex vivo* inhibition of GSK3α/β enhances the efficacy of adoptive T cell transfer in these models mirroring PD1 inhibition (49).

## Th17 Polarization Depends on GSK3β

The discovery of Th17 subset of CD4+ T lymphocytes, distinct from Th1 and Th2 cells was followed by exploration of their role in the tumor microenvironment (50). Th17 cells originate from naive CD4+ T cells that are exposed to TGFβ and IL6 or IL23, express the RAR orphan receptor gamma (RORγ) transcription factor and secrete IL17A, IL17F, IL21, IL22, and IL23. Additionally, Th17 cells can both originate from and also transform to Treg cells in a process known as Th17-Treg plasticity. Importantly, Th17 cells promote tumor growth in some models, while in others the effect is inhibitory. Dependence on context partially explains the Th17 paradox with TGFβ inducing an immunosuppressive, whereas IL23 an inflammatory phenotype on Th17 cells (51).

Polarization of CD4+ T cells into Th17 cells depends on active GSK3β *in vitro* and *in vivo* (52). Intriguingly, Th17 cells have 10-fold higher levels of GSK3β compared to other types of cells. Mechanistically, inhibition of GSK3 prevents IL6 production as well as STAT3 activation which are necessary steps for generation of Th17 cells. In disease models of *F. tularensis* pneumonia and experimental autoimmune encephalomyelitis (EAE), inhibition of GSK3β with lithium reduces the numbers of IL17A+CD4+ and IFNγ+CD4+ but spares Tregs in the lungs and spinal cords of mice, respectively. In both models, disease requires functional Th17 cells. In the EAE model, pre-treatment with lithium prevents development of the autoimmune phenotype and treatment of established EAE attenuates severity of symptoms. Notably, recruitment of CD8+ cells was reduced in the EAE model but was not affected in the *F. tularensis* pneumonia model. The role of GSK3β inhibition in Th17 cells in the tumor microenvironment is less well-studied.

## GSK3β Inhibition Enhances Treg Function

Tregs are important negative regulators of the adaptive immune response; they prevent autoimmune disease and maintain homeostasis (9, 10). They express CD4 and CD25, the high affinity receptor for interleukin 2 (IL-2). They also express the cytotoxic T-lymphocyte associated protein 4 (CTLA4) which binds to and induces endocytosis of B7-1 and B7-2, thereby antagonizes CD28 and blocks antigen presentation (9). The forkhead box P3 (FOXP3) transcription factor induces the expression of genes that promote Treg stability and immune suppressive function. GSK3α/β is among the genes identified as positive regulators of FOXP3 in an siRNA screen (53). Interestingly, pharmacological inhibition of GSK3β in Tregs enhances their suppressive function and limits the turnover of FOXP3 (53). GSK3β inhibition enhances the survival of islet transplant *in vivo* (54). Although this was shown with one inhibitor only, results are intriguing as they point out a possible immunosuppressive effect of GSK3β inhibitors in the tumor microenvironment.

## WNT/β Catenin and PI3K/AKT Pathways Are Associated With Cold Tumors

In a landmark study, Spranger et al. reported that the WNT/β catenin pathway is inversely correlated with T cell infiltration in melanoma tumors (55). The association was subsequently shown to occur in bladder cancer as well (4). Mechanistically, stabilization of β catenin and binding of the latter to the ATF3 promoter inhibits CCL4 expression. The chemokine CCL4, along with CCL3, CXCL1, and CXCL2 are ligands for the receptor CCR5 found in the cell surface of BATF3 positive dendritic cells. Activation of WNT/β catenin pathway inhibits the expression of these chemokines and prevents BATF3 positive dendritic cells from infiltrating the tumor (4, 55). In the absence of dendritic cells, CD8 positive T lymphocytes also fail to migrate in the tumor resulting in a “cold” or non-inflamed tumor microenvironment. Likewise, PTEN loss and PI3K/AKT pathway activation induce CCL2 and VEGF expression and inhibit infiltration of T lymphocytes in melanoma tumors (56). Although these studies did not look into the role of GSK3β directly, it is noteworthy that both WNTt/β catenin and PI3K/AKT pathways are activated by GSK3β inhibition.

Additionally, inhibition of GSK3β reduces interleukin 6 (IL-6) and increases interleukin 10 (IL-10) levels in lipopolysaccharide models of inflammation (57–59). IL-6 mediates an inflammatory reaction in the tumor microenvironment while IL-10 is immunosuppressive and associated with an M2 macrophage profile. Importantly, GSK3β inhibition in memory T cells induces IL-10 levels, while the effect on proliferation is minimal compared to naïve T cells. In this context, memory T cells adopt a regulatory/immunosuppressive role, as supernatants from memory T cell cultures with prior inhibition of GSK3β, limit the proliferation of receptor T lymphocytes in a IL-10 dependent fashion (58). Collectively, these data illustrate the multifaceted effects of GSK3β on the immune system as potentially both promoting and suppressing aspects of anti-tumor immunity.

## Discussion

A growing body of literature supports a multifaceted role for GSK3β in the immune tumor microenvironment. This is not a surprise as GSK3β is the busiest kinase in the cell based on the number of known and predicted substrates, many of which are key regulators of the immune response to tumors. Publication bias exists to some degree as there are either reports of GSK3β inhibitors as positive regulators of the immune response against tumors or negative regulators of the immune response in autoimmune conditions. In both contexts, GSK3β inhibitors are suggested as putative treatments. Both suppressing and enhancing immunity functions are possible for GSK3β. In fact, this has been shown in diverse models and comes as no surprise given the diversity of GSK3β substrates. Figure 2D summarizes the possible effects of GSK3β inhibitors on the immune system in the tumor microenvironment.

With this is mind, it is difficult to predict whether clinical GSK3β inhibition will enhance or suppress immunity against tumors. Further study of GSK3β regulation is required to define the context where GSK3β can be beneficial and support design of more precise drugs, such that target pro-tumorigenic while sparing the anti-tumorigenic functions of GSK3β.

## Author Contributions

AD and KS have contributed to the conception or design of the work as well as the acquisition, analysis or interpretation of studies included in this review. AD has drafted and KS revised the manuscript. Both authors approve publication of the content and agree to be accountable for all aspects of the work in ensuring that questions related to the accuracy or integrity of any part of the work are appropriately investigated and resolved.

## Conflict of Interest

AD has received honoraria by Roche/Genentech for a non-CME educational activity, not related to the content of this review. The remaining author declares that the research was conducted in the absence of any commercial or financial relationships that could be construed as a potential conflict of interest.
